# Trust in scientists and doctors: The roles of faith, politics, education and gender

**DOI:** 10.1177/09636625251386562

**Published:** 2025-11-16

**Authors:** Steven David Pickering, Martin Ejnar Hansen, Han Dorussen, Jason Reifler, Thomas J. Scotto, Yosuke Sunahara, Dorothy Yen

**Affiliations:** 1 University of Amsterdam, Netherlands; 2 Brunel University London, UK; 3 University of Essex, UK; 4 University of Southampton, UK; 5 University of Strathclyde, UK; 6 Kobe University, Japan; 7 Brunel University London, UK

**Keywords:** education, ethnicity, ideology, medical doctors, religion, scientists, trust in science, trust

## Abstract

This article examines trust in science in England, focusing on variation across demographic and ideological groups. Using survey data from 11,173 respondents, we compare trust in two domains, medical doctors and scientists, to explore whether predictors operate similarly across these professional groups. We find higher education is associated with greater trust, while right-wing political orientation predicts lower trust. Religious affiliation also matters, with some faith groups reporting lower trust relative to the non-religious baseline. Gender differences emerge as well, particularly in trust in medical doctors. Respondents selecting ‘Prefer not to say’ on the religion item report significantly lower trust in both doctors and scientists, consistent with a broader privacy-motivated disclosure style. Our results highlight the importance of considering not just overall levels of trust in science, but variation across education, ideology, religion and gender, and they suggest that trust in doctors and trust in scientists, while related, are not interchangeable.

## 1. Introduction

For evidence-based policy-making to be successfully implemented, society needs to trust the experts involved. The World Health Organization rates pandemics and climate change as the two major current threats to the planet. Addressing these threats relies heavily on science, from developing vaccines and treating illnesses to scientists working to mitigate climate change. When there are low levels of trust, or outright scepticism towards these areas the threat towards society is high (Goldenberg, 2023; Rutjens et al., 2022). However, the level of trust in science is not uniform across all subpopulations of society. Exploring how trust varies across society is vital for maintaining public confidence in science and ensuring compliance with policies and support for research. In this regard, trust and scepticism go hand in hand as concepts when examining this topic, despite their complex nature.

Existing research has highlighted the multifaceted nature of trust, a concept shaped by a variety of individual and conceptual factors (see Citrin and Stoker, 2018; Levi and Stoker, 2000). Similarly, specific trust, such as trust in government or the prime minister is shown to have an impact in how the public develop their attitudes towards trust in science (Resnick, 2022). Just as a person’s ideological views can shape their trust in experts and influence how they respond to messages that contradict their beliefs (Weingart, 2023), trust in political figures can affect public attitudes towards science. Overall, this has implications for how scientific and medical advice given by experts is perceived by the public and their general trust in science.

A further complicating element comes into play when strongly held personal beliefs clash with the scientific advice, for instance, when it comes to religious belonging (e.g. Catto et al., 2023). Within the different faiths there are varying levels of acceptance and engagement with science and medicine. For many religions there is no controversy with on the one hand listening to medical advice based on scientific achievement, while on the other hand seeking spiritual guidance from a particular religion. But this is not always the case. Some scholars have attempted to understand the impact of religiousness on medical outcomes (e.g. Wester et al., 2023). In this article, we specifically examine the connection between religious belonging and trust in scientists and medical doctors. We study this using a data set collected in England over nearly 2 years allowing us to include religious affiliation of individual respondents as a predictor variable.

Overall, there are high levels of trust in both medical doctors and scientists. When it comes to religious belonging we find a small number of faiths have their supporters associated with lower levels of trust towards both scientists and medical doctors. We also note that women have less trust in medical doctors than men, but are not significantly different from men when it comes to trust in scientists. Our findings corroborate existing research in highlighting the need for understanding subgroup differences in understanding levels of trust and in turn science scepticism. In addition, our findings can inform how public sector organisations communicate science-based initiatives, particularly when addressing groups with lower trust in the professionals behind these efforts.

While prior studies have examined trust in science in the United Kingdom, they often focus either on general institutional trust or domain-specific scepticism (e.g. vaccine hesitancy or climate change beliefs). Much of the comparative literature, meanwhile, relies heavily on US data, where political and religious dynamics differ markedly from those in the United Kingdom (Hornsey et al., 2018; Rutjens et al., 2022). Our study builds on this body of work by using high-frequency, large-sample survey data collected monthly across 18 months in England to explore trust in medical doctors and scientists: two of the most visible representatives of science in everyday life.

We focus particularly on three predictors that have received significant attention elsewhere but remain under-explored in the UK context: education, political ideology, and religious affiliation. While religion is often cited as a source of scepticism in science communication literature, empirical analyses of how specific religious affiliations affect trust in science within secular contexts like England are rare. This study thus contributes both by testing these relationships and by offering a detailed descriptive and multivariate breakdown of trust patterns across key demographic and ideological groups.

While our study examines several demographic and ideological predictors of trust in science, we place particular emphasis on religious affiliation, given its increasing importance in literature on science scepticism and vaccine hesitancy. We distinguish between the broader construct of religion, which includes belief systems and practices, and the narrower variable of religious affiliation (self-reported group identity) which we use in our analysis. Although religious affiliation does not capture the full complexity of religiosity, it provides an empirically tractable proxy for identifying how members of different faith groups may vary in their trust towards science and medicine. As we discuss below, we also explore variation between faith groups rather than treating religion as a single category, which allows for a more nuanced analysis.

In the next section, we explore the concept of trust – both broadly and in the context of science – along with the factors influencing it, before outlining our hypotheses. We then detail our data and methodology, followed by descriptive results and multivariate analysis. Finally, we discuss our findings in relation to existing literature, acknowledge the limitations of our study, and conclude with key remarks.

## 2. Trust and scepticism

Trust is central for understanding the relationship between individuals and institutions. Understanding individuals’ confidence in the government and political institutions can shed light on their trust in policies and their willingness to comply (Levi and Stoker, 2000). The concept of trust can be delineated between general and specific trust. The former refers to the levels of trust in political institutions at the societal level. This form of trust, shaped by social experiences, societal trends, and cultural norms, is often regarded as essential for fostering citizens’ connections with the political system through participation and cooperation (Rothstein and Stolle, 2008; van der Meer and Ouattara, 2019). This becomes especially important for ensuring that citizens are willing to accept collective decisions that are not aligned with their own personal interests.

The concept of specific trust is very much based on personal experience and knowledge about a particular institution (Citrin and Stoker, 2018; Levi and Stoker, 2000). It is a concept based on the individual’s values and expectations through their perceptions of competence, fairness and integrity. It is also a concept where repeated exposure to positive interactions is likely to increase trust, while failures might erode such trust. As such, specific trust can be seen as dynamic (Hetherington, 1998; Keele, 2007).

This underscores the importance of examining how individuals engage with science and how their experiences influence the development and evolution of trust in it. For instance, Pickering et al. (2024) argued that different experiences with policing had an influence on how much trust particular groups expressed towards the police. While Dorussen et al. (2024) established that negative experiences in the form of waiting times in the British National Health Service did not impact the levels of trust expressed towards the NHS. Viskupič et al. (2022) found that trust in government increased the uptake of the COVID-19 vaccine. It is generally accepted that trust in the delivery of healthcare services is extremely important (Calnan and Rowe, 2007; Gille et al., 2015; Rowe and Calnan, 2006), and the more research to better understand this relationship the better (Gille et al., 2021). Much of this research focuses on trust in the particular institutions and the services they provide and there is generally a strong relationship between the experience of healthcare and the level of trust (Kumlin and Rothstein, 2005). Yet, whether the trust also aligns towards the particular individuals working with the healthcare institution is a slightly different matter. This is also the case when we compare this level of trust in healthcare with the levels of trust in scientists. Medical doctors are often the primary representatives of science that individuals encounter, making them a reasonable proxy for trust in science when examining the relationship between independent variables and trust in science.

It is clear that trust and science scepticism are complex concepts. Trust in science often hinges on the perceived trustworthiness of scientists, which is influenced by political and demographic factors (Sarathchandra and Haltinner, 2020). As noted by Rutjens and van der Lee (2020), most of the knowledge we currently have about science scepticism is based on studies from the United States and the particular polarised nature of that country’s politics. However, as noted by Rutjens et al (2022), there are numerous factors influencing science scepticism, whether in terms of climate change or vaccine hesitancy. These factors include education (Motta, 2018), ideology (Hornsey et al., 2018; Rekker, 2021), and religious affiliation (Rutjens et al., 2018). Interestingly, in studies from the United States there is variation across domains with ideology being closely related to climate change, but not vaccines, while religiosity is more related to vaccine hesitancy (Hornsey et al., 2018; Rutjens et al., 2018).

It is also acknowledged that when it comes to religiousness the United States is an outlier among Western countries, highlighting the necessity to understand the issue in settings where religion plays a less prominent role. There are some exceptions to this US-bias; Rutjens et al (2022), for example, conducted a large-scale comparative study, albeit with small samples for each of the 24 countries included. One of the oft cited factors when it comes to lack of trust in science is related to religion. There is a growing body of research which shows a link between vaccine hesitancy and religion (Hansen and Pickering, 2024; Mao et al., 2024; Rutjens et al., 2018; Tiwana and Smith, 2024). Historically this is not new (Grabenstein, 2013). Interestingly, Rutjens et al (2022) and Hansen and Pickering (2024) also show that political views might also be influential for vaccine hesitancy, and Viskupič and Wiltse (2023) also look at government trust as having an impact. There is also recent evidence that vaccine hesitancy and trust in science are strongly connected (Scheitle and Corcoran, 2021).

## 3. Hypotheses

Building on this literature, we propose the following hypotheses to explore how demographic and ideological factors influence trust in medical doctors and scientists.

As Vaupotič et al. (2021) argue, in a modern society, individuals cannot achieve complete scientific literacy themselves; they must depend on the scientific credentials of others. This is built on trust and exposure, and individuals who have attended university will be more accustomed to deferring to the scientific knowledge of other people. Some existing work has empirically demonstrated a link between levels of education and perceptions of the *competence* of scientists (e.g. Heyerdahl et al., 2023). However, we are specifically interested in *trust*. Therefore, we hypothesise:

H1: Respondents with university education have higher levels of trust in medical doctors and scientists.

There is a strong expectation in the literature that people who identify as being on the right-wing of politics will tend to have lower levels of trust in doctors and scientists. However, much existing research has either been conducted exclusively in the highly polarised setting of the United States (e.g. Chido-Amajuoyi et al., 2023; Li and Qian, 2022) or has been conducted based on analyses of equally polarised data (see the exploration of Twitter data by Altenmüller et al., 2024). Accordingly:

H2: The more right-wing the respondent, the lower level of trust in medical doctors and scientists.

Finally, much recent research has found a link between religion and vaccine hesitancy (Hansen and Pickering, 2024; Mao et al., 2024; Rutjens et al., 2018; Tiwana and Smith, 2024). We argue that one of the foundations for this is lower levels of trust among people with religious identity. As such:

H3: Respondents affiliated with a religion display lower levels of trust in medical doctors and scientists.

In the next section, we present the data used to test the hypotheses and the methodology used.

## 4. Data and methodology

The data used in this article come from a series of monthly surveys run from December 2022 to June 2024 in England by the survey company YouGov using a non-probability quota sampling approach to approximate national representativeness in England. All respondents were unique individuals, with no panel overlap across waves. While quota-based samples are not fully randomised, they are widely used in UK academic research and public policy, particularly for tracking public opinion trends over time. As such, we qualify our use of the term ‘representative’ to mean that the sample reflects national distributions on key demographics such as gender, age, region, and education. However, limitations around generalisability remain, particularly for smaller subgroups such as some religious minorities. These concerns are addressed in our discussion of study limitations. YouGov’s survey methodology is widely used in both academic and policy settings in the United Kingdom, and their approach has been benchmarked against other public opinion measures such as the British Election Study. Replication data and code are available through the Harvard Dataverse, at https://doi.org/10.7910/DVN/Z7A1GC.

A total of 11,173 respondents are included in the data and all respondents are unique observations. In the survey, respondents were asked a number of questions, here specifically with regards to trust in medical doctors and scientists which we use as proxies for trust in science. The questions were the following:

- Using a scale of 1 to 7 where 1 means ‘not at all’ and 7 means ‘completely’, how much do you trust medical doctors?
- Using a scale of 1 to 7 where 1 means ‘not at all’ and 7 means ‘completely’, how much do you trust scientists working at universities?

The choice of prompting respondents specifically with scientists *working at universities* was to avoid the respondents’ views towards private companies by specifically asking them to only consider those working at universities. The two variables above are our dependent variables.

For independent variables we include a number of socio-demographic variables, such as whether the respondent is female, their age, and whether they have completed university education. We also include measures for trust and political views, for example, their level of general trust, their levels of specific trust in the government and in the prime minister, their self-placement on a left-right scale, and finally their self-reported religious affiliation. This was based on YouGov’s standard panel item on whether respondents belong to any particular religion. Response options include ‘No, I do not regard myself as belonging to any particular religion’, a series of ‘Yes’ options, with the name of the denomination or faith, and a ‘Prefer not to say’ option. Respondents were also able to skip the item altogether. We retain both Prefer not to say (PNS) and skipped as separate categories.

General trust is based on whether respondents feel that, in general, most people can be trusted. As with the trust in medical doctors and scientists questions (above), our other trust questions were also asked on a 7-point scale. Including these questions as control variables is important to ensure that our expected associations are present when correcting for the presence of important variables such as general social trust (Hetherington, 1998; Newton and Zmerli, 2011; van der Meer and Ouattara, 2019). The left-right political ideology scale used in the survey ranges from 0 (left-wing) to 11 (right-wing), with respondents self-placing along this continuum. In the UK context during the survey period (2022–2024), the left of the scale generally corresponds to parties such as Labour, the Greens, and Liberal Democrats, while the right end maps onto support for the Conservative Party and parties like Reform UK. Unlike in more polarised two-party systems, UK voters often exhibit a broader ideological range and greater volatility in party support, so this self-placement captures ideological tendencies rather than strict partisanship. Comparatively it is established that there is a connection between ideological self-placement and trust (Nielsen and Petersen, 2023) and the inclusion of this variable as a control is important for considering limitations for our findings.

The descriptive statistics of our variables can be found in Table 1. There is a slight bias towards more female respondents than male. Mean general trust levels fall to the middle of a 7-point scale, with notably lower levels of trust in the government and prime minister. On the left-right political spectrum, our respondents fall broadly into a third on the left, a third in the middle and a third on the right.

**Table 1.** Descriptive statistics.

Variable	Value
Age (Mean)	49.96
Age (Min)	18
Age (Max)	98
Age (SD)	17.71
Men (%)	45.45
Women (%)	54.55
Ethnic minority (count)	1486.00
Ethnic minority (%)	13.56
Less than University (%)	63.70
University (%)	36.30
Left (%)	34.41
Centre (%)	34.64
Right (%)	30.96
Trust General (Mean)	3.60
Trust General (Min)	1
Trust General (Max)	7
Trust General (SD)	1.59
Trust Gov (Mean)	2.71
Trust Gov (Min)	1
Trust Gov (Max)	7
Trust Gov (SD)	1.53
Trust PM (Mean)	2.76
Trust PM (Min)	1
Trust PM (Max)	7
Trust PM (SD)	1.66
Sample Size (N)	11173

To assess the predictors of trust in medical doctors and scientists, we estimate a series of regression models that build cumulatively. The initial models exclude religious affiliation to establish baseline associations. We then introduce religious affiliation to test for general effects compared with non-religious respondents, and finally reframe the analysis using Church of England respondents as the reference category to explore variation across religious subgroups. This structure allows us to assess both the overall and relative effects of religious identity, while keeping other key demographic and attitudinal predictors constant.

Table 2 breaks down our respondent base by religion. As can be seen, just over a half of our respondents have no religion. Of the religious, the single biggest category is the Church of England. It should be noted that over 3 per cent of our respondents preferred not to declare their religion, and over 4 per cent of respondents chose to skip this question.

**Table 2.** Respondents’ religion.

Religion	Count	Percentage
No religion	5765	51.6
Church of England	2521	22.56
Roman Catholic	788	7.05
Other Christian	88	0.79
Methodist	184	1.65
Baptist	78	0.7
Judaism	79	0.71
Hindu	89	0.8
Islam	171	1.53
Other	310	2.77
Orthodox	86	0.77
Pentecostal	102	0.91
Evangelical	97	0.87
Prefer not to say	353	3.16
Skipped question	462	4.13

## 5. Analysis

**Figure 1.**
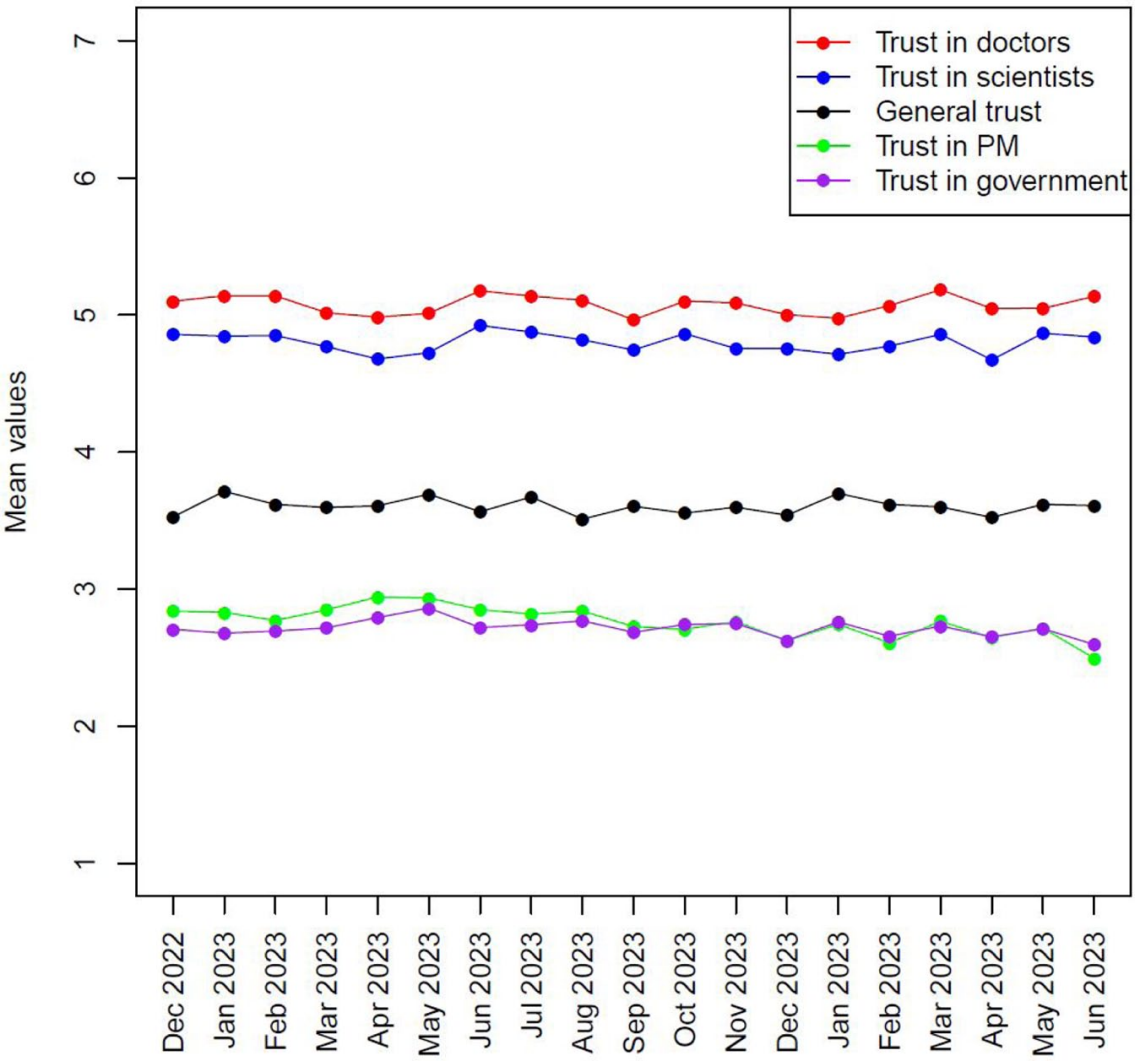
Trust in Doctors and Scientists. Note: for colour figure please see online version.

Our analysis was conducted using ordinary least squares regression, but before we present our multivariate results we first show the differences between the levels of trust in medical doctors and scientists among the respondents as depicted in Figure 1. Trust towards medical doctors varies between 5.0 and 5.1 on a 7-point scale across the waves, while trust towards scientists varies between 4.8 and 4.9 on the same scale. In other words, we should not expect to see markedly different outcomes in how science is viewed whether we use trust in medical doctors or trust in scientists as proxies. Interestingly, baseline trust of doctors and scientists is notably higher than our other trust categories (general trust, trust in the Prime Minister, and trust in the government).

In Table 3, we present the results of three sets of regressions. Models 1 and 2 do not include a measure for religion, while models 3 and 4 compare respondents’ religious affiliation with the reference category of ‘non-religious’. Models 5 and 6 exclude those who are non-religious and instead compare the various religious groups with the Church of England respondents as the reference category.

**Table 3.** Regression models.

	(1) Trust doctors	(2) Trust scientists	(3) Trust doctors	(4) Trust scientists	(5) Trust doctors	(6) Trust scientists
Intercept	4.48^***^ (0.06)	4.34^***^ (0.06)	4.52^***^ (0.06)	4.38^***^ (0.06)	4.48^***^ (0.10)	4.30^***^ (0.10)
Age	0.00^***^ (0.00)	-0.00^***^ (0.00)	0.00^***^ (0.00)	-0.00^***^ (0.00)	0.00^***^ (0.00)	-0.00^***^ (0.00)
Women	-0.18^***^ (0.02)	-0.02 (0.02)	-0.19^***^ (0.02)	-0.02 (0.02)	-0.17^***^ (0.04)	0.03 (0.04)
Ethnic	-0.27^***^ (0.04)	-0.26^***^ (0.04)	-0.20^***^ (0.04)	-0.17^***^ (0.04)	-0.21^***^ (0.06)	-0.18^***^ (0.06)
University	0.09^***^ (0.03)	0.34^***^ (0.03)	0.08^***^ (0.03)	0.34^***^ (0.03)	0.05 (0.04)	0.29^***^ (0.04)
Left-right	-0.13^***^ (0.01)	-0.17^***^ (0.01)	-0.13^***^ (0.01)	-0.17^***^ (0.01)	-0.11^***^ (0.01)	-0.15^***^ (0.01)
General trust	0.19^***^ (0.01)	0.21^***^ (0.01)	0.19^***^ (0.01)	0.21^***^ (0.01)	0.16^***^ (0.01)	0.17^***^ (0.01)
Trust government	0.15^***^ (0.01)	0.14^***^ (0.01)	0.15^***^ (0.01)	0.14^***^ (0.01)	0.16^***^ (0.02)	0.14^***^ (0.02)
Trust PM	0.05^***^ (0.01)	0.07^***^ (0.01)	0.05^***^ (0.01)	0.08^***^ (0.01)	0.05^***^ (0.02)	0.10^***^ (0.02)
Church of England			0.03 (0.03)	-0.04 (0.03)		
Roman Catholic			0.00 (0.05)	-0.14^***^ (0.05)	-0.01 (0.05)	-0.09 (0.06)
Other Christian			-0.16 (0.13)	-0.18 (0.14)	-0.19 (0.14)	-0.12 (0.14)
Methodist			0.12 (0.09)	0.11 (0.10)	0.10 (0.10)	0.16 (0.10)
Baptist			-0.04 (0.14)	-0.22 (0.15)	-0.05 (0.15)	-0.15 (0.15)
Judaism			-0.08 (0.14)	-0.11 (0.15)	-0.10 (0.14)	-0.06 (0.15)
Hindu			-0.19 (0.14)	-0.18 (0.15)	-0.19 (0.15)	-0.13 (0.15)
Islam			-0.24^**^ (0.10)	-0.39^***^ (0.11)	-0.23^**^ (0.11)	-0.33^***^ (0.12)
Other			-0.33^***^ (0.07)	-0.32^***^ (0.08)	-0.33^***^ (0.08)	-0.25^***^ (0.08)
Orthodox			-0.32^**^ (0.14)	-0.12 (0.14)	-0.33^**^ (0.14)	-0.08 (0.15)
Pentecostal			-0.02 (0.13)	-0.49^***^ (0.13)	-0.02 (0.13)	-0.45^***^ (0.14)
Evangelical			0.25^*^ (0.13)	-0.08 (0.13)	0.25^*^ (0.13)	-0.01 (0.14)
Prefer not to say			-0.46^***^ (0.07)	-0.61^***^ (0.08)	-0.47^***^ (0.08)	-0.56^***^ (0.08)
Skipped question			0.07 (0.06)	0.04 (0.06)	0.08 (0.07)	0.10 (0.07)
R^2^	0.15	0.19	0.15	0.20	0.15	0.17
Adj. R^2^	0.15	0.19	0.15	0.20	0.15	0.17
Num. obs.	10842	10842	10842	10842	5202	5202

Standard errors in parentheses, ***p < 0.01, **p < .05, *p < .1. For models 3–4, the reference category for religion is ‘no religion’, for models 5–6 it is ‘Church of England’.

*** *p* < .01; **p < .05; *p < .1.

We posed three hypotheses, the first of which relates to education and in five of the six models respondents with a university education have significantly higher levels of trust in doctors and scientists than those without a university education. The exception is towards doctors when the non-religious respondents are excluded. Here the coefficient is insignificant. Our second hypotheses related to ideology, and in all six models the more right-wing the respondent the less trust there is in science, as measured by our proxies.

Our third hypothesis was related to religious affiliation. Here the results are mixed. Some religious affiliation, Islam and Other Faiths (non-Christian) are associated with a significantly lower level of trust in science, as proxied through both scientists and medical doctors. Orthodox Christians have lower levels of trust in doctors, while Pentecostal respondents have lower levels of trust in scientists. In one model, Roman Catholic respondents have lower levels of trust in scientists: this is the case when the non-religious are used as the baseline (model 4), but not when the Church of England is used as the baseline (model 6).

Curiously, the single group with the strongest effect is the ‘Prefer not to say’ (PNS) category. As noted in the Data & Methodology section, the religion item offered ‘No, I do not regard myself as belonging to any particular religion’, a set of ‘Yes – [denomination/faith]’ categories, and ‘Prefer not to say’. Respondents could also skip the item altogether. In all four religion models (models 3–6), the 353 respondents selecting PNS report about half a point lower trust (on a 1–7 scale) in both doctors and scientists. By contrast, the 462 respondents who skipped the question show no difference in trust relative to either the ‘no religion’ or Church of England baselines. We interpret the PNS finding as evidence of a privacy-motivated disclosure style rather than simple item skipping. Sensitive items typically elicit higher nonresponse rates, as respondents avoid revealing information they consider private or stigmatising (Plutzer, 2019). Furthermore, item refusal is often systematically related to question sensitivity and respondent traits (Silber et al., 2021). Privacy framing and salience also significantly affect willingness to disclose sensitive information, with respondents more likely to select PNS options when the perceived sensitivity of the item is heightened (Joinson et al., 2008; Murdoch, 2014). In contexts where ‘Prefer not to say’ is offered, this response choice may signal that respondents are withholding identifiers, possibly as a way to manage exposure or stigma (Warner et al., 2020). That PNS respondents remain less trusting once generalised trust is taken into account suggests selective institutional wariness: continued reticence in engagement even with trusted expertise.

We also note that belonging to an ethnic minority in the United Kingdom is associated with significantly lower levels of trust in science across the board. The three trust measures we have included as controls, that is, general trust and two forms of specific trust are associated with higher levels of trust in science. People who trust other people, or institutions, tend to have more trust in science. Interestingly we see a significant but different connection when it comes to age, the older the respondent the more trust in doctors, but the less in scientists. Also, for women there is an effect, but only for doctors who are significantly less trusted by women than by men, and no effect is established when it comes to scientists.

Overall our findings provide several different perspectives. First of all, there are differences across subgroups depending on which proxy is used, that is, medical doctors and scientists are not the same entirely. In a more secular nation like the United Kingdom, it is also problematic to talk about religion in general, some faiths have a general sceptical view towards science, while others again depend on how science is perceived. This has strong implications for policy-makers, as our findings suggest that the ‘sender’ of a scientific message might well have influence on how it is received among particular subgroups. However, we also acknowledge that our results do not include the various interactions that one might wish for, that is, between the various independent variables, especially the religious groups. For this our sample, which is nationally representative, does not have enough respondents to allow for in-depth interactions. A more targeted sample would be needed here. We also fully acknowledge that our assumption that trust in medical doctors and scientists can be taken as proxies for trust in science, and in reverse, science scepticism, might be a step too far. However, we do note that our findings of variation in factors being associated with trust across subgroups are similar to findings across other settings.

## 6. Limitations

Although our dataset includes over 11,000 respondents, and thus allows for high statistical power, we do not assume that large-N automatically guarantees substantive or inferential robustness. We employ regression models to assess relationships between predictors and trust in science, but we are careful to interpret statistical significance alongside effect sizes and contextual plausibility. Given the non-probability sampling and the limitations in subgroup sample sizes (especially for smaller religious groups), we refrain from making overly generalised claims and instead interpret our findings as indicative of patterns rather than definitive conclusions.

We also recognise several additional limitations. First, the use of quota-based sampling via an online panel limits our ability to generalise to the full population, particularly with respect to harder-to-reach groups. Second, while we focus on religious affiliation as a key predictor, this variable does not capture broader dimensions of religiosity such as belief intensity, practice, or spirituality. Third, while our overall sample is large, the size of certain subgroups restricts our ability to explore interaction effects or conduct more granular analysis of religious and ideological intersections. Fourth, our research design permits associational rather than causal conclusions. Establishing causality would require a very different design, which would also face challenges related to sample size, particularly for the smaller subgroups. Finally, the surveys were conducted during the post-COVID period, a time when trust in science and public health institutions remained politically and culturally salient, potentially shaping responses in ways not fully captured in our model. We encourage future studies to build on our findings using probability-based samples, experimental research designs, more granular religious and ideological measures, and targeted recruitment of specific subgroups.

## 7. Conclusion

Our study sheds light on the complicated relationship between trust, demographic factors and science scepticism, using trust in medical doctors and scientists as proxies for trust in science. We find that while trust in science remains generally high, significant variation exists across demographic and ideological lines. Education, political orientation, and religious affiliation are key factors influencing trust levels.

Respondents with higher levels of education have greater levels of trust in medical doctors and scientists. This aligns with existing literature, demonstrating the role of education in fostering trust. Political ideology also plays a significant role, with right-wing respondents exhibiting lower levels of trust in science. This reflects the case of the more polarised setting of the United States. Religious affiliation further complicates the picture, as some faith groups report lower trust in scientists and medical professionals, highlighting the complex relationship between personal beliefs and perceptions of expertise. Trust in scientists and trust in doctors can be seen as proxies for trust in *science*, but the two are not exactly the same, as is shown in our observations on religion.

These findings offer several practical implications for improving trust in science and medicine. First, public health communicators should consider not just the content of messaging but also the identity of the messenger. Trusted community figures – such as local medical professionals, religious leaders, or grassroots organisers – may serve as more effective conduits of scientific information in communities with lower baseline trust. Second, message framing should be adapted to match the values and communication norms of specific groups. For example, narrative-based messaging or testimonial formats may resonate more with religious audiences than purely data-driven appeals. Finally, communications should be tailored to specific domains of trust: as our findings show, trust in doctors and scientists is not always interchangeable, and this distinction should inform outreach strategies.

Our findings can also be situated within the broader international literature on public trust in science. While research from the United States often highlights the strong influence of political ideology – particularly right-wing populism – on trust in science, our results suggest that in the UK context, ideology plays a role but interacts with different institutional and cultural dynamics. Compared with other European countries (e.g. Heyerdahl et al., 2023), levels of trust in scientists in England remain relatively high, but subgroup differences – especially among ethnic minorities and certain religious communities – are more pronounced. This aligns with recent comparative research (e.g. Rutjens et al., 2022) which finds that trust in science varies not only by individual factors but by national context, shaped by historical and institutional trust patterns. Our study contributes to this growing body of comparative work by offering a detailed analysis of trust predictors in a relatively secular, post-pandemic European setting.

We also acknowledge the limitations of our study. While nationally representative, our sample lacked sufficient depth to explore complex interactions between religion and political ideology. In addition, our reliance on proxies for trust in science calls for caution in generalising the findings. Future research should aim to address these gaps, potentially through targeted sampling and expanded measures of trust. Ultimately, our research underscores the importance of understanding subgroup differences in trust and scepticism towards science. One striking example is the group of respondents who selected ‘Prefer not to say’ when asked about religious affiliation. Their consistently lower trust in doctors and scientists suggests that privacy-related disclosure styles may themselves mark a distinct dimension of trust in science. This is an area future work should explore. In a rapidly changing world, where science is pivotal in addressing global challenges, fostering trust across diverse populations is increasingly important.

## Supplemental Material

sj-docx-1-pus-10.1177_09636625251386562 – Supplemental material for Trust in scientists and doctors: The roles of faith, politics, education and gender Supplemental material, sj-docx-1-pus-10.1177_09636625251386562 for Trust in scientists and doctors: The roles of faith, politics, education and gender by Steven David Pickering, Martin Ejnar Hansen, Han Dorussen, Jason Reifler, Thomas J. Scotto, Yosuke Sunahara and Dorothy Yen in Public Understanding of Science
