# Supplementary material for: Trust in scientists and doctors: The roles of faith, politics, education and gender
Source: Public Underst Sci. 2025 Nov 16;35(3):352–66. doi: 10.1177/09636625251386562 (PMC12999988; doi:10.1177/09636625251386562)
Supplement: sj-docx-1-pus-10.1177_09636625251386562 – Supplemental material for Trust in scientists and doctors: The roles of faith, politics, education and gender [file sj-docx-1-pus-10.1177_09636625251386562.docx]

**Survey Questions for:**

Trust in Scientists and Doctors: The Roles of Faith, Politics, Education and Gender

**Authors:**

Steven David Pickering, University of Amsterdam (corresponding author) – [s.d.pickering@uva.nl](mailto:s.d.pickering@uva.nl), ORCID 0000-0002-1357-2994
Martin Ejnar Hansen, Brunel University London – [martin.hansen@brunel.ac.uk](mailto:martin.hansen@brunel.ac.uk), ORCID 0000-0002-3637-208X
Han Dorussen, University of Essex – [hdorus@essex.ac.uk](mailto:hdorus@essex.ac.uk), ORCID 0000-0002-3458-0555
Jason Reifler, University of Southampton – [j.reifler@soton.ac.uk](mailto:j.reifler@soton.ac.uk), ORCID 0000-0002-1116-7346
Thomas Scotto, University of Strathclyde – [tom.scotto@strath.ac.uk](mailto:tom.scotto@strath.ac.uk), ORCID 0000-0003-4801-6821
Yosuke Sunahara, Kobe University – [sunahara@people.kobe-u.ac.jp](mailto:sunahara@people.kobe-u.ac.jp), ORCID 0009-0001-0759-1478
Dorothy Yen, Brunel University London – [dorothy.yen@brunel.ac.uk](mailto:dorothy.yen@brunel.ac.uk), ORCID 0000-0003-1129-9653

Code and replication data are available from the Harvard Dataverse, at:
<https://doi.org/10.7910/DVN/Z7A1GC>

When users first set up their accounts on YouGov, they are asked a variety of demographic questions. For this research, we used the following:

In what year were you born?

[Based on this, YouGov provides the respondent’s age, not the year they were born.]

YouGov also asks:

Are you...?

Female

Male

YouGov asks respondents about their religion. They are given the following options (and do not see the numbers):

1 No, I do not regard myself as belonging to any particular religion.

2 Yes - Church of England/Anglican/Episcopal

3 Yes - Roman Catholic

4 Yes - Presbyterian/Church of Scotland

5 Yes - Methodist

6 Yes - Baptist

7 Yes - United Reformed Church

8 Yes - Free Presbyterian

9 Yes - Brethren

10 Yes - Judaism

11 Yes - Hinduism

12 Yes - Islam

13 Yes - Sikhism

14 Yes - Buddhism

15 Yes - Other

16 Prefer not to say

17 Yes – Orthodox Christian

18 Yes - Pentecostal (e.g. Assemblies of God, Elim Pentecostal Church, New Testament Church of God, Redeemed Christian Church)

19 Yes - Evangelical – independent/non-denominational (e.g. FIEC, Pioneer, Vineyard, Newfrontiers)

98a Skipped

99a Not Asked

[None of our respondents had the 99a (Not Asked) code; therefore, they were all asked.]

Our questions on trust in scientists and medical doctors were fielded every month from December 2022 to June 2024. Below, we report the questions used in this research. Survey questions which were not included in this research have been replaced with “…”.

This survey is on a variety of topics, and the results will be used to inform our clients.

Your YouGov Account will be credited with 50 points for completing the survey.

We have tested the survey and found that, on average it takes around 15 minutes to complete. This time may vary depending on factors such as your Internet connection speed and the answers you give.

Please click the forward button below to continue.

Thanks for taking our survey. We are going to start by asking you to indicate how much you trust various people and institutions.

…

Generally speaking, would you say that most people can be trusted, or that you can’t be too careful in dealing with people?

1 - You cannot be too careful

2

3

4

5

6

7 - Most people can be trusted

Using a scale of 1 to 7 where 1 means "not at all" and 7 means "completely", how much do you trust each of the following:

1 - Not at all 2 3 4 5 6 7 - Completely

NATO

The BBC

The European Union

The Liberal Democrats

The World Health Organisation (WHO)

Scientists working at universities

The military

The United Nations

The Labour Party

The Conservative Party

Medical doctors

[The order of the above was randomised.]

…

In politics people sometimes talk of “left” and “right”. Where would you place yourself on this scale, where 0 means the left and 10 means the right?

Scale element with the minimum value labelled as "Left", and the maximum value labelled as "Right"

Left

0

1

2

3

4

5

6

7

8

9

10

Right

…

Thank you for answering this survey. All questions were part of an academic experiment. Please click the button below to complete the survey.
